# A case of Mycoplasma pneumoniae‐induced rash and mucositis with recent influenza vaccination

**DOI:** 10.1002/ski2.459

**Published:** 2024-09-16

**Authors:** Vani Agarwal, Georgie Gamble, Alexander Amphlett, Neil P. Patel

**Affiliations:** ^1^ Department of Dermatology Imperial College Healthcare NHS Trust London UK; ^2^ Department of Histopathology Imperial College Healthcare NHS Trust London UK

## Abstract

A 33‐year‐old female presented with coryzal symptoms, facial swelling, severe haemorrhagic stomatitis, blistering oral mucositis, conjunctival injection and a sparse targetoid rash on the back and face, requiring admission to hospital. She had received the seasonal influenza vaccination 3 days prior to feeling unwell. Differential diagnosis included erythema multiforme major (EMM) secondary to the influenza vaccine or Mycoplasma pneumoniae‐induced rash and mucositis (MIRM). Oropharyngeal swabs were negative on PCR for cutaneous viruses and *Mycoplasma pneumoniae* (MP). A skin biopsy from a targetoid lesion on the body showed full thickness epidermal necrosis with epidermal–dermal clefting, numerous civatte bodies and a moderate perivascular infiltrate of lymphocytes and plasma cells in the papillary dermis. She was successfully treated with oral prednisolone and azithromycin. Following discharge from hospital, the paired serological testing for MP returned positive, confirming a diagnosis of MIRM. Our case highlights the difficulties in detecting MP as two diagnostic methods yielded different results, and so we advocate performing both MP PCR and serology to maximise the yield and speed of diagnosis. Secondly, our case highlights the clinical challenge in differentiating MIRM from EMM or Stevens–Johnson syndrome, particularly if there is a potential drug trigger (in our case the influenza vaccine), as all these conditions can feature a severe mucositis with often indistinguishable histological findings. Correct diagnosis of MIRM is important for appropriate and timely administration of anti‐MP antibiotic therapy to facilitate recovery and minimise complications.

## Graphical abstract

A 33‐year‐old female presented with coryzal symptoms, facial swelling, severe haemorrhagic stomatitis, blistering oral mucositis, conjunctival injection, and a sparse targetoid rash on the back and face, requiring admission to hospital. Oropharyngeal swab PCR was negative but serology was positive for *Mycoplasma pneumoniae* (MP), yielding a diagnosis of Mycoplasma pneumoniae‐induced rash and mucositis (MIRM). We discuss the challenges involved in detecting MP using the available tests and the difficulties in clinically distinguishing MIRM from erythema multiforme major and Stevens–Johnson syndrome.

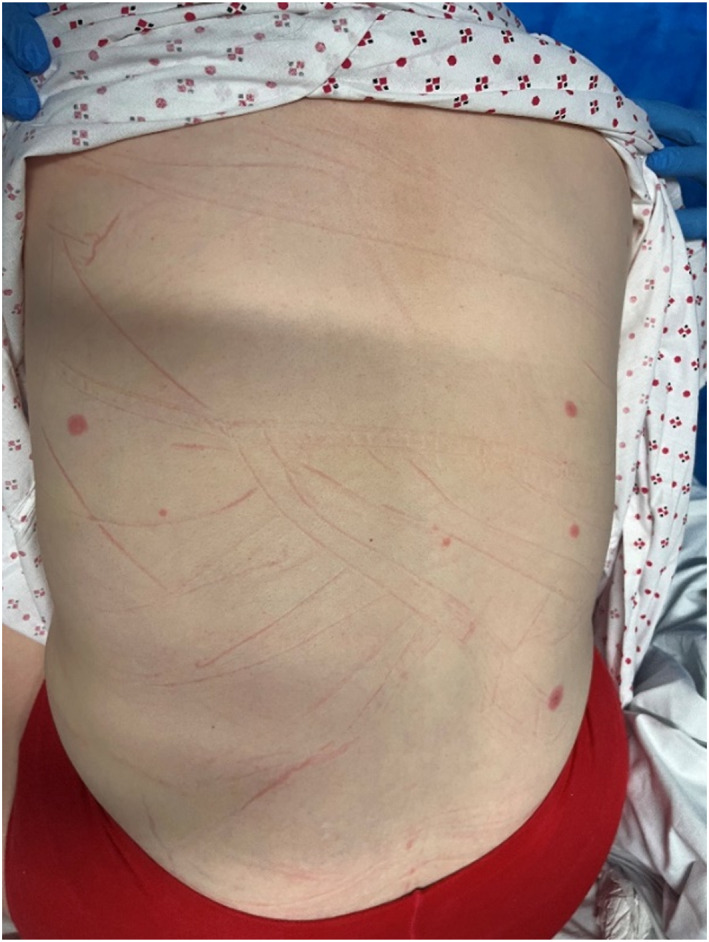

## 1 CASE REPORT

**FIGURE 1:**
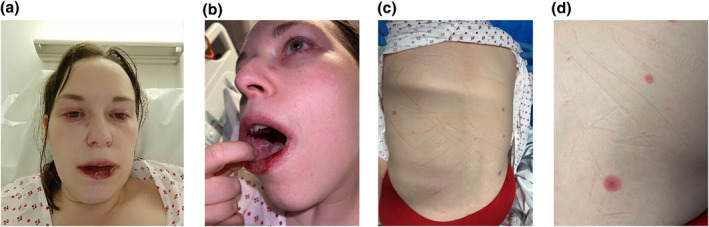
Clinical photographs demonstrating (a) facial swelling, periorbital inflammation, conjunctival injection, a targetoid lesion on the nasal sidewall, and haemorrhagic crusting of the lips; (b) stomatitis and (c, d) sparse targetoid lesions on the back.

A 33‐year‐old female was admitted to hospital with a 2‐day history of facial swelling and oral ulceration. This was preceded by 4 days of fever, headache, sore throat and myalgia. She had received the seasonal influenza vaccination (which she was accustomed to having annually due to living with a vulnerable partner) 3 days prior to feeling unwell. She had no medical history, no recent travel history, was not taking any regular medications and was not pregnant. Examination revealed facial swelling, severe haemorrhagic erosions and crusting affecting the lips, and severe blistering mucositis affecting the buccal mucosa and hard palate. Two days after presentation, she developed periorbital swelling, conjunctival injection, inflammation around the nostrils and sparse targetoid lesions on the back and face (Figure 1).

**FIGURE 2:**
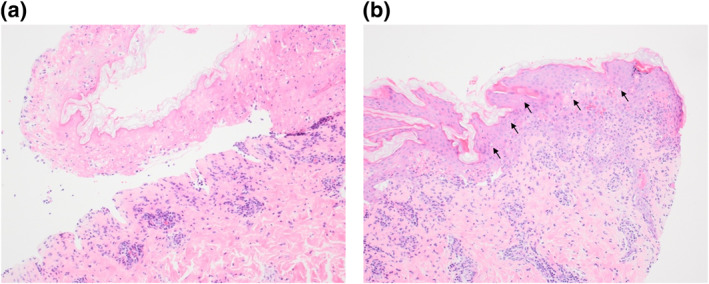
Skin histopathology demonstrates (a) subepidermal cleft with full thickness epidermal necrosis. The papillary dermis shows a moderate perivascular infiltrate of lymphocytes and plasma cells (haematoxylin and eosin [H&E] stain; magnification ×100). (b) The non‐necrotic epidermis shows frequent civatte bodies (black arrows) and lymphocyte exocytosis (H&E ×100).

Initial investigations revealed a highly elevated c‐reactive protein of 340 mg/L and a mild lymphopenia of 0.8 × 10^9^/L. Blood culture was negative and chest radiograph was normal. Oropharyngeal swabs were negative on PCR for herpesviruses, respiratory viruses and *Mycoplasma pneumoniae* (MP). Paired serology for MP was done at presentation and again 7 days later. Other causes of atypical pneumonia (Legionella, *Coxiella burnetii*, *Chlamydia psittacci*) were excluded by urinary antigen testing and serological tests. A skin biopsy taken from a targetoid lesion on the back showed full thickness epidermal necrosis with epidermal–dermal clefting, numerous civatte bodies and a moderate perivascular infiltrate of lymphocytes and plasma cells in the papillary dermis (Figure 2).

Differential diagnosis at this stage included erythema multiforme major (EMM) secondary to the influenza vaccine or Mycoplasma pneumoniae‐induced rash and mucositis (MIRM). Treatment was commenced to cover both possibilities with oral prednisolone 40 mg daily and oral azithromycin. She responded rapidly to treatment and was discharged after 6 days on a weaning regime of oral prednisolone. The ocular symptoms were self‐limiting and vision was not threatened, and so ophthalmology review was not required.

Two weeks after discharge, the paired MP serology was reported as being compatible with a recent infection; total antibodies 85.0 AU/mL (>13.3 AU/mL is positive) and immunoglobulin M > 27.0 AU/mL (>12.3 AU/mL is positive). The incubation period for MP infection is 2–3 weeks,^1^ indicating that the infective trigger in this case preceded the influenza vaccine, although we cannot exclude the possibility that immune stimulation by the vaccine contributed to the clinical presentation.

## 2 DISCUSSION

MIRM was described as a distinct entity from erythema multiforme and Stevens–Johnson syndrome (SJS) in 2015.^2^ There is a predilection for males and paediatric patients. Typical clinical features include prodromal symptoms preceding the rash, severe mucosal involvement (usually two or more sites), often minimal skin changes^3^ and a quick resolution with antibiotics and oral corticosteroids. Histology of MIRM has been shown to have a toxic epidermal necrolysis‐like pattern.^4^

Diagnostic tests for MP include culture, molecular tests and serology. Molecular tests such as PCR have a high specificity and sensitivity with timely results^5^; however, they are operator‐dependent and positivity often depends on the bacterial load at the time of testing. Serology has a high sensitivity when performed on paired acute (from week one of symptoms) and convalescent (a further 1–2 weeks later) samples.^1^, ^5^ Disadvantages of serological testing include the potential for patient unavailability for the convalescent sample if discharged from hospital and long laboratory processing times leading to diagnostic delay.

Our case highlights the difficulties in detecting MP as two diagnostic methods yielded different results. Relying on molecular PCR testing alone would have led to a missed diagnosis of MIRM in our patient. Serology did confirm the diagnosis, but delayed laboratory results limited the impact on our patient's care. Therefore, we advocate performing both MP PCR and serology to maximise the yield and speed of diagnosis.

Secondly, our case highlights the clinical challenge in differentiating MIRM from EMM or SJS, particularly if there is a potential drug trigger (in our case the influenza vaccine^6^), as all these conditions can feature a severe mucositis. This clinical conundrum can be compounded by often indistinguishable histological findings between the conditions. Correct diagnosis of MIRM is important for appropriate and timely administration of anti‐MP antibiotic therapy to facilitate recovery and minimise complications. In our case, the diagnosis of MIRM meant that we were able to advise the patient to continue receiving the seasonal influenza vaccination every year. Had a diagnosis of MIRM not been established, the patient would have been advised to avoid the influenza vaccination lifelong, potentially putting her vulnerable partner at risk from influenza infection.

## CONFLICT OF INTEREST STATEMENT

The authors declare no conflicts of interest.

## AUTHOR CONTRIBUTIONS

**Vani Agarwal**: Conceptualization (lead); supervision (supporting); writing—original draft (lead). **Georgie Gamble**: Writing—original draft (supporting); writing—review and editing (supporting). **Alexander Amphlett**: Data curation (equal); formal analysis (equal); methodology (equal); resources (equal). **Neil P. Patel**: Supervision (lead); writing—review and editing (lead).

## ETHICS STATEMENT

Not applicable.

## PATIENT CONSENT

Written patient consent for publication was obtained.

## Data Availability

Data sharing is not applicable to this article as no new data were created or analysed in this study.
